# *Vibrio vulnificus* MO6-24/O Lipopolysaccharide Stimulates Superoxide Anion, Thromboxane B_2_, Matrix Metalloproteinase-9, Cytokine and Chemokine Release by Rat Brain Microglia *in Vitro*

**DOI:** 10.3390/md12041732

**Published:** 2014-03-26

**Authors:** Alejandro M. S. Mayer, Mary L. Hall, Michael Holland, Cristina De Castro, Antonio Molinaro, Monica Aldulescu, Jeffrey Frenkel, Lauren Ottenhoff, David Rowley, Jan Powell

**Affiliations:** 1 Department of Pharmacology, Chicago College of Osteopathic Medicine, Midwestern University, 555 31st Street, Downers Grove, IL 60515, USA; E-Mails: mhallx@midwestern.edu (M.L.H.); hollandmichaela@gmail.com (M.H.); monica.aldulescu@mwumail.midwestern.edu (M.A.); jfrenk25@gmail.com (J.F.); lottenhoff57@midwestern.edu (L.O.); 2 Biomedical Sciences Program, College of Health Science, Midwestern University, 555 31st Street, Downers Grove, IL 60515, USA; 3 Department of Chemical Sciences, University of Napoli, Via Cintia 4, 80126 Napoli, Italy; E-Mails: decastro@unina.it (C.D.C.); molinaro@unina.it (A.M.); 4 Department of Biomedical and Pharmaceutical Sciences, College of Pharmacy, University of Rhode Island, 7 Greenhouse Road, Kingston, RI 02881, USA; E-Mail: drowley@mail.uri.edu; 5 Department of Epidemiology and Preventive Medicine, University of Maryland School of Medicine, 10 Pine St, Baltimore, MD 21201, USA; E-Mail: jpowell@shire.com; 6 Shire Human Genetic Therapies, 300 Shire Way, Lexington, MA 02421, USA

**Keywords:** LPS, *Escherichia coli*, *Vibrio vulnificus*, rat microglia, cytokine, chemokine, superoxide, thromboxane, metalloproteinase, neuroinflammation, MMP-9

## Abstract

Although human exposure to Gram-negative *Vibrio vulnificus* (*V. vulnificus*) lipopolysaccharide (LPS) has been reported to result in septic shock, its impact on the central nervous system’s innate immunity remains undetermined. The purpose of this study was to determine whether *V. vulnificus* MO6-24/O LPS might activate rat microglia *in vitro* and stimulate the release of superoxide anion (O_2_^−^), a reactive oxygen species known to cause oxidative stress and neuronal injury *in vivo*. Brain microglia were isolated from neonatal rats, and then treated with either *V. vulnificus* MO6-24/O LPS or *Escherichia coli* O26:B6 LPS for 17 hours *in vitro*. O_2_^−^ was determined by cytochrome C reduction, and matrix metalloproteinase-2 (MMP-2) and MMP-9 by gelatinase zymography. Generation of cytokines tumor necrosis factor alpha (TNF-α), interleukin-1 alpha (IL-1α), IL-6, and transforming growth factor-beta 1 (TGF-β1), chemokines macrophage inflammatory protein (MIP-1α)/chemokine (C-C motif) ligand 3 (CCL3), MIP-2/chemokine (C-X-C motif) ligand 2 (CXCL2), monocyte chemotactic protein-1 (MCP-1)/CCL2, and cytokine-induced neutrophil chemoattractant-2alpha/beta (CINC-2α/β)/CXCL3, and brain-derived neurotrophic factor (BDNF), were determined by specific immunoassays. Priming of rat microglia by *V. vulnificus* MO6-24/O LPS *in vitro* yielded a bell-shaped dose-response curve for PMA (phorbol 12-myristate 13-acetate)-stimulated O_2_^−^ generation: (1) 0.1–1 ng/mL *V. vulnificus* LPS enhanced O_2_^−^ generation significantly but with limited inflammatory mediator generation; (2) 10–100 ng/mL *V. vulnificus* LPS maximized O_2_^−^ generation with concomitant release of thromboxane B_2_ (TXB_2_), matrix metalloproteinase-9 (MMP-9), and several cytokines and chemokines; (3) 1000–100,000 ng/mL *V. vulnificus* LPS, with the exception of TXB_2_, yielded both attenuated O_2_^−^ production, and a progressive decrease in MMP-9, cytokines and chemokines investigated. Thus concentration-dependent treatment of neonatal brain microglia with *V. vulnificus* MO6-24/O LPS resulted in a significant rise in O_2_^−^ production, followed by a progressive decrease in O_2_^−^ release, with concomitant release of lactic dehydrogenase (LDH), and generation of TXB_2_, MMP-9, cytokines and chemokines. We hypothesize that the inflammatory mediators investigated may be cytotoxic to microglia *in vitro*, by an as yet undetermined autocrine mechanism. Although *V. vulnificus* LPS was less potent than *E. coli* LPS *in vitro*, inflammatory mediator release by the former was clearly more efficacious. Finally, we hypothesize that should *V. vulnificus* LPS gain entry into the CNS, it would be possible that microglia might become activated, resulting in high levels of O_2_^−^ as well as neuroinflammatory TXB_2_, MMP-9, cytokines and chemokines.

## 1. Introduction

*Vibrio vulnificus* (*V. vulnificus*) is a virulent halophilic motile Gram-negative bacterium present in marine, estuarine and aquaculture warm water environments worldwide [1,2]. *V. vulnificus* may infect humans through contaminated seafood or skin wounds, causing gastroenteritis, necrotic skin infections, primary septicemia, with fatality rates reported to exceed 50% [2,3], and meningitis [4,5]. Although combination antimicrobial therapy of *V. vulnificus* meningitis has resulted in effective treatment [5], antibiotic resistance in *V. vulnificus* is a definite concern [3,6]. Clinical and environmental sources of *V. vulnificus*, which include pathogenic as well as non-pathogenic strains, are currently divided into three biotypes [7,8]. Interestingly, because the biotype of *V. vulnificus* isolated from cerebrospinal fluid in meningoencephalitis and meningitis cases was not characterized, a correlation between clinical and environmental *V. vulnificus* biotypes and potential brain infections in humans remains presently undetermined [4,5]. 

Research into the chemistry and immunotoxicology of *V. vulnificus* lipopolysaccharides (LPS) was initiated more than two decades ago with the isolation of *V. vulnificus* LPS [9]. Previous studies have investigated both the O-polysaccharide or O-antigen of the LPS molecule, which are responsible for immunogenicity [10], as well as the lipid A moiety which is associated with Gram-negative septic shock [11]. Several studies on the pathogenicity of *V. vulnificus* LPS in mice and rats have revealed that it may be pyrogenic and cause cardiovascular injury [10,12,13], which may progress to septic shock and high mortality [10,14], pathological conditions which have been shown to be affected by chronic iron overload, estrogen and low-density lipoprotein [15,16,17,18]. *V. vulnificus* Biotype 1, strain MO6-24/O, which has been shown to be lethal to mice [14] and to induce both interleukin-6 mRNA and tumor necrosis factor-α (TNF-α) release from human peripheral blood mononuclear cells [19], was used in this study. To our knowledge, there is no report in the literature that has determined the effect of *V. vulnificus* LPS on brain microglia, the main cell type involved in neuroinflammation [20].

In humans, Gram-negative infections and release of LPS in the circulation may result in a systemic inflammatory response that contributes to sepsis and refractory septic shock [21] and may also impact the brain [22]. Furthermore, if LPS causes a pathological disruption of the blood-brain barrier (BBB) [23] or penetrates the brain via regions where the BBB is defective, it may activate brain microglia [24]. When microglia are activated by LPS via interaction with the CD14 receptor and Toll-like receptor 4 [22,25], inflammatory mediators are released including reactive oxygen species, e.g., O_2_^−^ [26,27], which may cause neuronal injury [28], and progressive neurodegeneration [29,30]. To our knowledge no studies have been completed to determine the effect of *V. vulnificus* LPS on brain microglia O_2_^−^ generation.

The purpose of this investigation was to test the hypothesis that *in vitro* treatment of neonatal rat microglia with *V. vulnificus* MO6-24/O LPS might stimulate release of O_2_^−^, a reactive oxygen species hypothesized to be associated with brain injury [31]. Together with our preliminary communications [32,33,34], the current study provides experimental support for our working hypothesis, namely that *V. vulnificus* LPS primes rat brain microglia *in vitro* for O_2_^−^ generation. Furthermore, O_2_^−^ generation appeared to be concomitant with the release of several pro-inflammatory mediators, namely thromboxane B_2_ and matrix metalloproteinases, as well as several cytokines and chemokines. 

## 2. Results

### 2.1. Effect of V. vulnificus LPS on Rat Brain Microglia O_2_^−^ Generation

**Figure 1:**
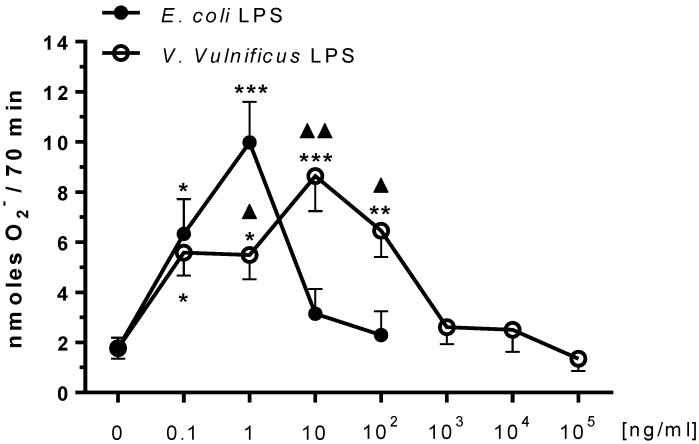
The effect of *E. coli* and *V. vulnificus* lipopolysaccharide (LPS) on rat microglia phorbol 12-myristate 13-acetate (PMA)-stimulated O_2_^−^ release. Neonatal rat microglia (2 × 10^5^ cells/well) were treated with *E. coli* LPS (0.1–100 ng/mL) or *V. vulnificus* LPS (0.1–10^5^ ng/mL) for 17 h *in vitro*, and then stimulated with PMA (1 μM) for 70 min. O_2_^−^ was determined as described in Experimental Section. Data expressed as nanomoles O_2_^−^ is the mean ± SEM of 13–14 independent experiments (*n*), each experiment with duplicate determinations. ** *P* < 0.01, *** *P* < 0.001 *versus* untreated control (0). ^▲^ *P* < 0.05, ^▲▲^ *P* < 0.01 *V. vulnificus* LPS *vs. E. coli* LPS.

Microglia reactive oxygen species generation has been reported to be associated with oxidative stress in chronic neurodegenerative diseases [35,36,37]. We have repeatedly observed that *E. coli* LPS pre-treatment primes rat microglia for agonist-stimulated O_2_^−^ generation *in vitro* [27,38]. As shown in Figure 1, untreated microglia release low levels of O_2_^−^ after phorbol 12-myristate 13-acetate (PMA) stimulation. However, when *E. coli* LPS-treated microglia were stimulated with PMA, O_2_^−^ generation was bell-shaped with maximal O_2_^−^ at 1 ng/mL LPS (9.98 ± 1.6 nmoles O_2_^−^, *n* = 14, *P* < 0.001), confirming our previous observations [27]. In contrast, when microglia were pre-treated with *V. vulnificus* LPS for 17 h, concentration-dependent O_2_^−^ release became maximal at 10 ng/mL *V. vulnificus* LPS (8.6 ± 1.4 nmoles O_2_^−^, *n* = 13, *P* < 0.001), and then progressively decreased. Thus *V. vulnificus* LPS appeared to be approximately a tenth less potent than *E. coli* LPS in stimulating O_2_^−^ production from microglia *in vitro*. 

### 2.2. Effect of V. vulnificus LPS on Rat Brain Microglia LDH Generation

In order to determine whether the progressive decrease in O_2_^−^ release shown in Figure 1 was caused by toxicity of *E. coli* or *V. vulnificus* LPS to microglia, we measured the presence of lactate dehydrogenase (LDH), a marker for cellular toxicity, in microglia tissue culture supernatants after the 17 h *in vitro* incubation [39]. 

**Figure 2:**
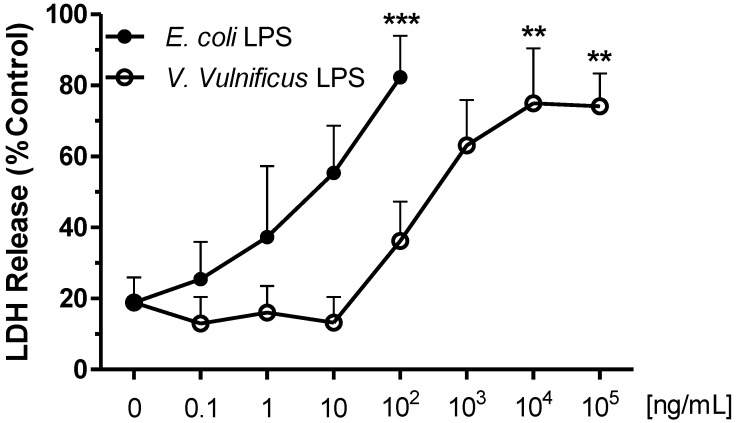
The effect of *E. coli* and *V. vulnificus* LPS on rat microglia lactate dehydrogenase (LDH) release.Neonatal rat microglia (2 × 10^5^ cells/well) were treated with either *E. coli* LPS (0.1–100 ng/mL) or *V. vulnificus* LPS (0.1–10^5^ ng/mL) for 17 h *in vitro*. LDH release was determined as described in Experimental Section. Data expressed as % of Triton X-100 0.1% treated microglia LDH release is the mean ± SEM of 12–13 independent experiments (*n*), each experiment with duplicate determinations. ** *P* < 0.01, *** *P* < 0.001 *versus* untreated control (0).

As shown in Figure 2, there was a dose-dependent increase in LDH release *in vitro* that paralleled the decrease in O_2_^−^ generation observed with increasing *E. coli* or *V. vulnificus* LPS concentrations*.* Thus in *E. coli* LPS-pretreated microglia, statistically significant LDH release was observed at 100 ng/mL LPS (73.6 ± 8.4% of control, *n* = 12, *P* < 0.001). In contrast, in *V. vulnificus* LPS-pretreated microglia, a maximum of 70.3 ± 16% of control LDH release was observed at 10^4^ ng/mL (*n* = 12, *P* < 0.01). 

### 2.3. Effect of V. vulnificus LPS on Rat Brain Microglia TXB_2_ Generation

**Figure 3:**
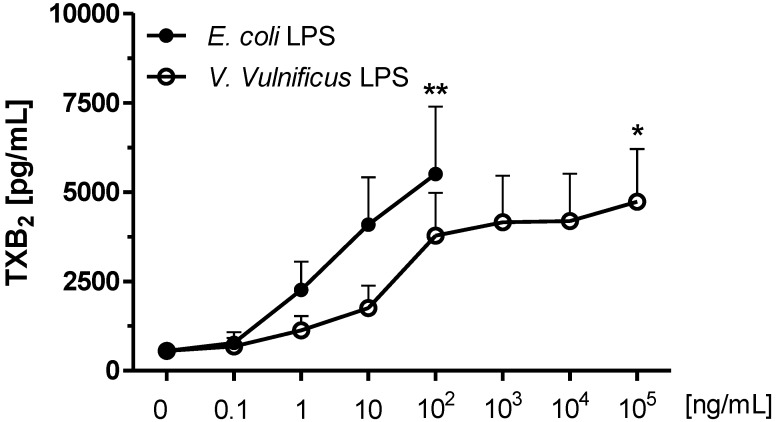
The effect of *E. coli* and *V. vulnificus* LPS on rat microglia thromboxane B_2_ (TXB_2_) release. Neonatal rat microglia (2 × 10^5^ cells/well) were treated with *E. coli* LPS (0.1–100 ng/mL) or *V. vulnificus* LPS (0.1–10^5^ ng/mL) for 17 h *in vitro*. TXB_2_ was determined as described in Experimental Section. Data expressed as TXB_2_ pg/mL is the mean ± SEM of seven independent experiments (*n*), each experiment with duplicate determinations. * *P* < 0.05, ** *P* < 0.01 *versus* untreated control (0).

Release of eicosanoids by activated microglia appears to play a significant role in neuroinflammation [26], and *E. coli* LPS-treated rat microglia generate TXB_2_ *in vitro* [27,40]. As depicted in Figure 3, unstimulated microglia released low levels of TXB_2_ (550 ± 200 pg/mL, *n* = 8). Confirming prior observations [27,38], when microglia were pre-treated with 100 ng/mL *E. coli* LPS, high concentrations of TXB_2_ were detected in the tissue culture supernates (5504.5 ± 1881 pg/mL TXB_2_, *n* = 7, *P* < 0.01), which was concomitant with attenuated O_2_^−^ generation (Figure 1), and enhanced LDH release (Figure 2). In contrast, *V. vulnificus* LPS-treated microglia TXB_2_ generation yielded a sigmoid curve, and became statistically significant at 10^5^ ng/mL LPS (4731 ± 1475 pg/mL TXB_2_ *n* = 7, *P* < 0.05), when there was minimal O_2_^−^ generation (Figure 1) and high LDH release (Figure 2). Thus, although *V. vulnificus* LPS appeared less potent than *E. coli* LPS in inducing a concentration-dependent TXB_2_ release from rat microglia *in vitro,* it was as effective as *E. coli* LPS, because maximal TXB_2_ release was of a similar magnitude. 

### 2.4. Effect of V. vulnificus LPS on Rat Brain Microglia MMP-2 and MMP-9 Generation

**Figure 4:**
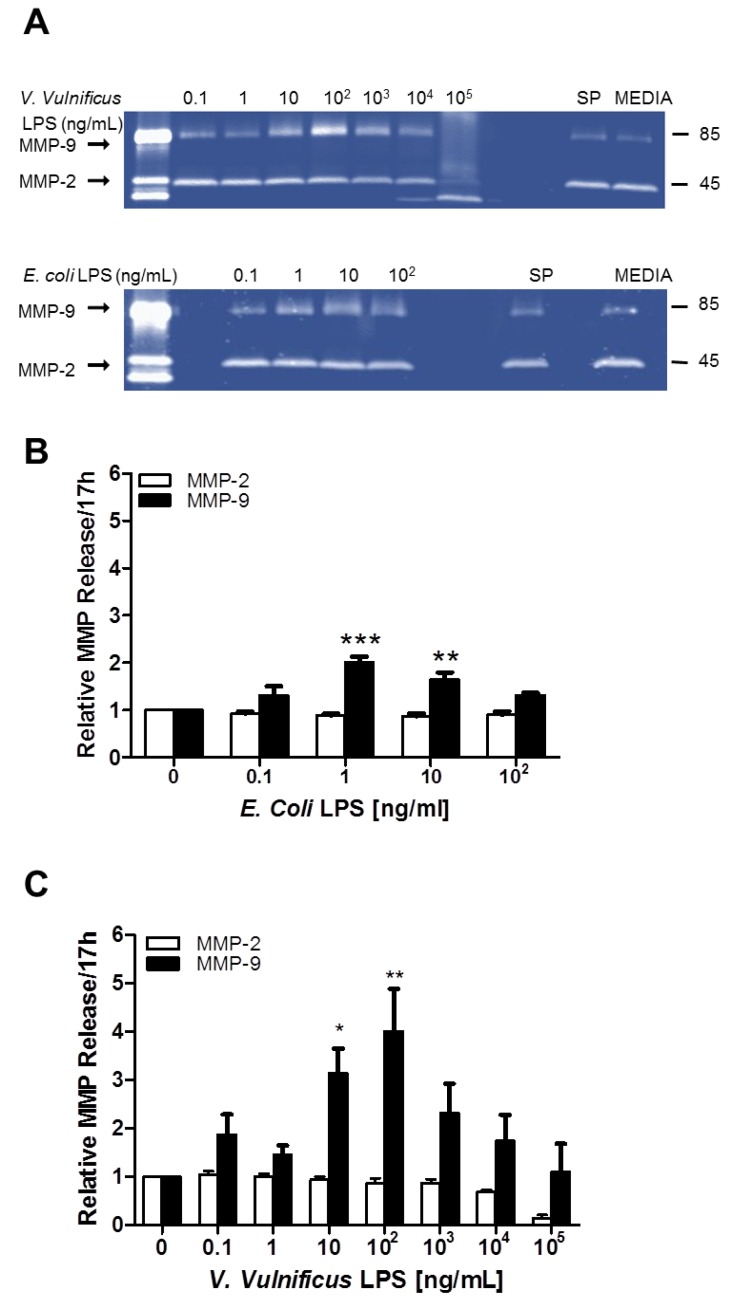
The effect of *E. coli* and *V. vulnificus* LPS on rat microglia matrix metalloproteinase-2 (MMP-2) and -9 release. Neonatal rat microglia (2 × 10^5^ cells/well) were treated with *E. coli* LPS (0.1–100 ng/mL) or *V. vulnificus* LPS (0.1–10^5^ ng/mL) for 17 h *in vitro*. As described in Experimental Section, MMP-2 and MMP-9 were determined by sodium dodecyl sulfate polyacrylamide gel electrophoresis (SDS-PAGE) zymography (panel **A**), and quantitated results for *E. coli* LPS (panel **B**) and for *V. vulnificus* LPS (panel **C**) are depicted in the bar graphs. Data is expressed as mean ± SEM of normalized MMP release from five independent experiments (*n*). * *P* < 0.05, ** *P* < 0.01, *** *P* < 0.001 *versus* untreated control (0).

Matrix metalloproteinases (MMP) released by activated microglia have been proposed to be proinflammatory in both sepsis and neuroinflammation [41,42]. We, and others, have observed MMP-2 and MMP-9 generation by *E. coli* LPS-stimulated rat microglia *in vitro* [27,43]. As shown in Figure 4, in *E. coli* LPS-stimulated microglia, MMP-9 levels were statistically significant at the following LPS concentrations (ng/mL): One (2.0 ± 0.12-fold, *n* = 5, *P* < 0.001), and 10 (1.64 ± 0.15-fold, *n* = 5, *P* < 0.01), respectively. Similarly, microglia stimulated with *V. vulnificus* LPS for 17 h demonstrated a concentration-dependent increase of MMP-9, but not of MMP-2, at the following LPS concentrations (ng/mL): 10 (3.1 ± 0.5- fold, *n* = 5, *P* < 0.05), and 100 (4.0 ± 0.9-fold, *n* = 5, *P* < 0.01). Thus while *V. vulnificus* LPS was a tenth less potent than *E. coli* LPS in inducing a concentration-dependent release of MMP-9, it was more effective than *E. coli* LPS, because maximal release MMP-9 was two-fold higher. 

### 2.5. Effect of V. vulnificus LPS On Rat Brain Microglia Cytokines IL-6, TNF-α and IL-1α Release

After a 17 h *in vitro* incubation, both *E. coli* and *V. vulnificus* LPS-treated microglia released the inflammatory cytokines IL-6, TNF-α, and IL-1α into the tissue culture supernates, in the following decreasing concentrations (pg/mL): IL-6 > TNF-α > IL-1α.

**Figure 5:**
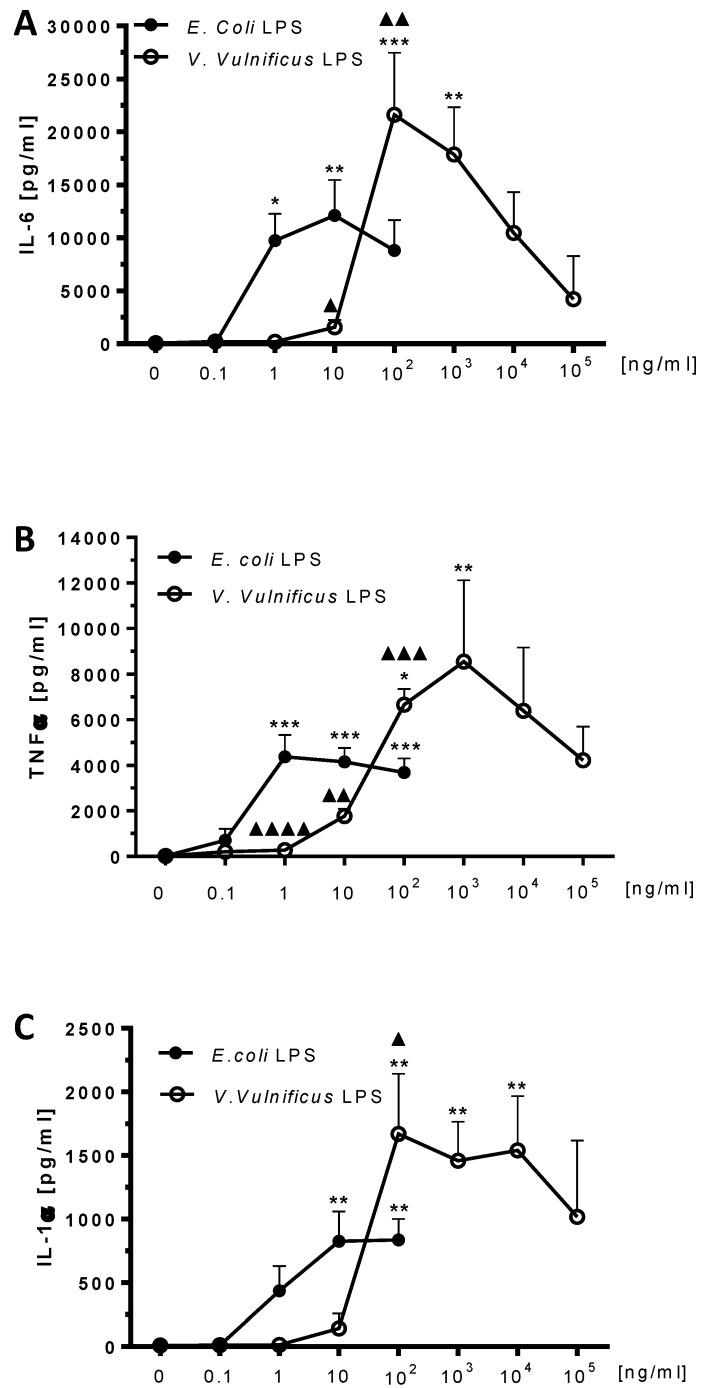
The effect of *E. coli* and *V. vulnificus* LPS on rat microglia cytokines IL-6, tumor necrosis factor-alpha (TNF-α) and interleukin-one alpha (IL-1α) release. Neonatal rat microglia (2 × 10^5^ cells/well) were treated with *E. coli* LPS (0.1–100 ng/mL) or *V. vulnificus* LPS (0.1–10^5^ ng/mL) for 17 h *in vitro*. IL-6 (Panel **A**), TNF-α (Panel **B**), and IL-1α (Panel **C**) were determined as described under Experimental Section. Data expressed as pg/mL is the mean ± SEM of values from four to six independent experiments (*n*), each experiment with duplicate determinations. * *P* < 0.05, ** *P* < 0.01, *** *P* < 0.001 *versus* untreated control (0). *^▲^ P* < 0.05, ^▲▲^ *P* < 0.01, ^▲▲▲^ *P* < 0.001, ^▲▲▲▲^ *P* < 0.0001 *V. vulnificus* LPS *vs. E. coli* LPS*.*

The cytokine IL-6 has been shown to play a role in the immune response, inflammation and hematopoiesis [44], and in neuroimmunomodulation in Alzheimer’s disease [45]. *E. coli* LPS has been reported to stimulate human [46], murine [47] and rat [43,48] microglia to release IL-6 *in vitro*. In our investigation, unstimulated microglia released low levels of IL-6 (71.7 ± 35 pg/mL, *n* = 4). As shown in Figure 5 (Panel A), IL-6 levels in *E. coli* LPS-stimulated microglia were significant at 1 ng/mL LPS (9733 ± 2539 pg/mL IL-6, *n* = 4, *P* < 0.05). In *V. vulnificus* LPS-stimulated microglia, IL-6 levels became statistically significant at 100 ng/mL (21,605 ± 5864 pg/mL IL-6, *n* = 4, *P* < 0.001). Thus, *V. vulnificus* LPS appeared to be a hundredth less potent than *E. coli* LPS in inducing concentration-dependent release of IL-6 from rat microglia *in vitro,* but was more efficacious than *E. coli* LPS, because maximal release of IL-6 was 78% higher. 

The cytokine TNF-α has been shown to play an important role in normal brain function as well as in neurodegenerative disease [49]. Treatment of human [46], murine [47,50], and rat microglia [27,48] with *E. coli* LPS *in vitro* resulted in TNF-α release. In our present investigation, unstimulated rat microglia released low levels of TNF-α (16.5 ± 7.9 pg/mL, *n* =6). As shown in Figure 5 (Panel B), in *E. coli* LPS-stimulated microglia, a concentration-dependent TNF-α release became statistically significant at 1 ng/mL LPS (4363 ± 961 pg/mL TNF-α, *n* = 5, *P* < 0.001), thus confirming our previous observations [27]. In contrast, in *V. vulnificus* LPS-stimulated rat microglia cells, TNF-α release was significant at 100 ng/mL, and peaked at 1000 ng/mL (8547 ± 3570 pg/mL TNF-α, *n* = 6, *P* < 0.01). Thus, similar to O_2_^−^ (Figure 1) and TXB_2_ (Figure 3), *V. vulnificus* LPS appeared to be a hundredth less potent than *E. coli* LPS in inducing TNF-α production from rat microglia *in vitro*, and yet *V. vulnificus* LPS was clearly more efficacious than *E. coli* LPS, because maximal TNF-α release was 132% higher. 

The cytokine IL-1α has been proposed as a key mediator in inflammatory neurodegeneration and neuronal injury [51,52]. *E. coli* LPS primes rat microglia to release IL-1α *in vitro* [53]. In our present study, unstimulated microglia released low levels of IL-1α (9.2 ± 5.1 pg/mL, *n* = 4). As shown in Figure 5 (Panel C), in *E. coli* LPS-stimulated microglia, IL-1α levels peak at 10 ng/mL (825.7 ± 234.6 pg/mL IL-1α, *n* = 4, *P* < 0.01). In contrast, in *V. vulnificus* LPS-stimulated microglia, IL-1α levels become statistically significant at 100 ng/mL (1669.7 ± 473.3 pg/mL IL-1α, *n* = 4, *P* < 0.01). Thus, although *V. vulnificus* LPS was a tenth less potent than *E. coli* LPS in inducing IL-1α generation from rat microglia *in vitro,* as observed for TNF-α (Figure 5, panel B) generation, the overall magnitude of the *V. vulnificus* LPS response was higher than *E. coli* LPS, with an increase in maximal release of IL-1α of 102%. 

### 2.6. Effect of V. vulnificus LPS on Rat Brain Microglia MIP-2/CXCL2, MIP-1α/CCL3, CINC-2α/β (CXCL3) and MCP-1/CCL2 Generation

*E. coli* and *V. vulnificus* LPS-treated microglia released several inflammatory chemokines into the tissue culture supernates after a 17 h *in vitro* incubation in the following decreasing concentrations (pg/mL): MIP-2/CXCL2 > MIP-1α/CCL3 > CINC-2α/β (CXCL3) > MCP-1/CCL2.

**Figure 6:**
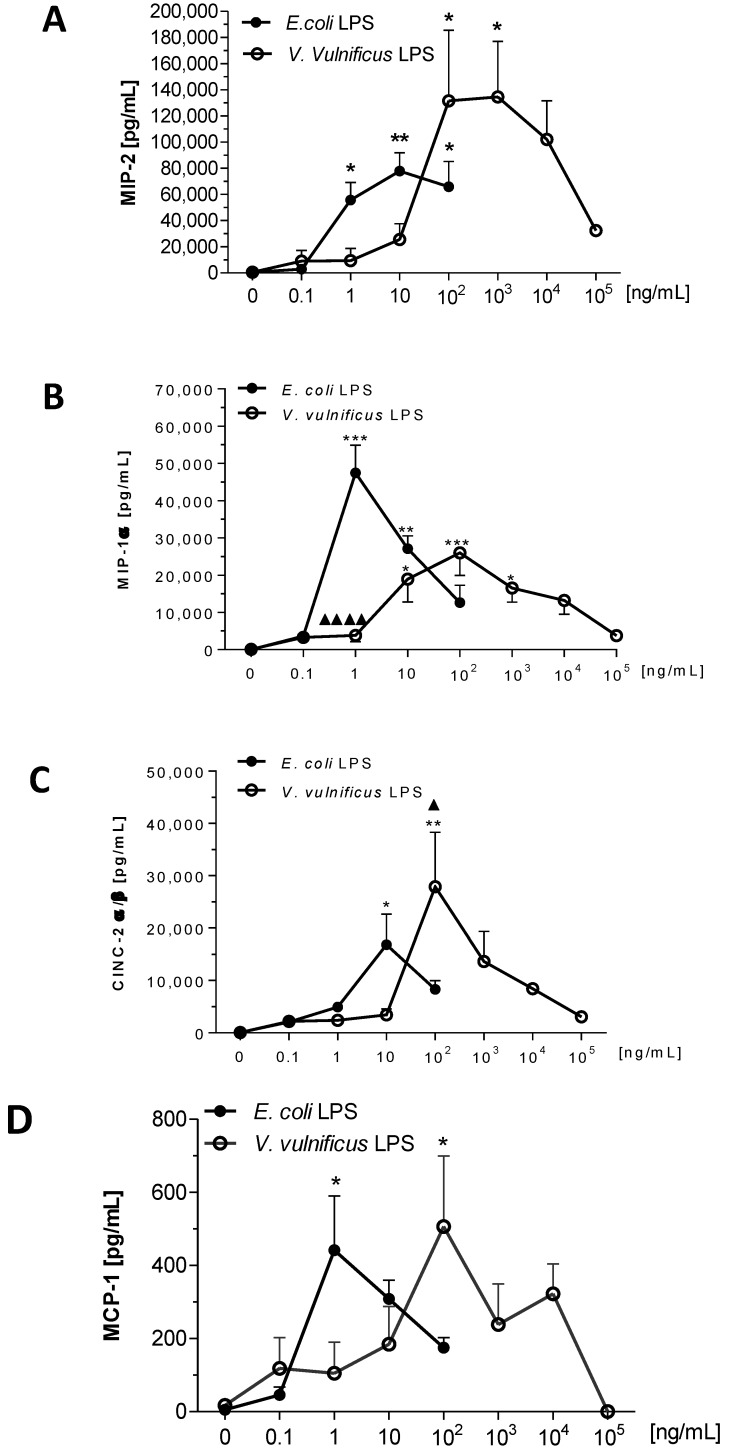
The effect of *E. coli* and *V. vulnificus* LPS on rat microglia chemokines macrophage inflammatory protein-2 (MIP-2)/chemokine (C-X-C motif) ligand 2 (CXCL2), MIP-1α/chemokine (C-C motif) ligand 3 (CCL3), CINC-2α/β/CXCL3 and monocyte chemotactic protein-1 (MCP-1)/CCL2 release. Neonatal rat microglia (2 × 10^5^ cells/well) were treated with *E. coli* (0.1–100 ng/mL) or *V. vulnificus* (0.1–10^5^ ng/mL) LPS for 17 h *in vitro*. MIP-2/CXCL2 (Panel **A**), MIP-1α/CCL3 (Panel **B**), CINC-2α/β/CXCL3 (Panel **C**) and MCP-1/CCL2 (Panel **D**) release was determined as described in Experimental Section. Data expressed as pg/mL is the mean ± SEM of two to four independent experiments (*n*), each experiment with duplicate determinations. * *P* < 0.05; ** *P* < 0.01; *** *P* < 0.001 *versus* untreated control (0). *^▲^ P* < 0.05, ^▲▲▲▲^ *P* < 0.0001 *V. vulnificus* LPS *vs. E. coli* LPS*.*

The chemokine MIP-2 or CXCL2, which is involved in neuroinflammation [54], has been reported to be released by LPS-treated murine [47,55] and rat [56] microglia *in vitro*. Unstimulated microglia released low levels of MIP-2 constitutively (560 ± 302 pg/mL, *n* = 3). As shown in Figure 6 (Panel A), MIP-2/CXCL2 levels in *E. coli* LPS-stimulated microglia rose after 0.1 ng/mL LPS, and were maximal at 10 ng/mL LPS (77,951 ± 13,927 pg/mL MIP-2/CXCL2, *n* = 3, *P* < 0.01). In *V. vulnificus* LPS-stimulated microglia, MIP-2/CXCL2 release progressively increased at 10 ng/mL LPS, and became statistically significant at 100 ng/mL (131,581 ± 54,023 pg/mL MIP-2/CXCL2, *n* = 3, *P* < 0.05). Thus, similar to the cytokines TNF-α, IL-1α and IL-6, *V. vulnificus* LPS was less potent than *E. coli* LPS in inducing concentration-dependent release of the chemokine MIP-2/CXCL2 *in vitro* but more efficacious because maximal release was 73% higher.

The chemokine MIP-1α or CCL3, involved in CNS inflammation [54] and in multiple sclerosis [57], has been reported to be generated by LPS-treated mouse [47,50], rat [58] and human microglia cells *in vitro* [59]. As shown in Figure 6 (Panel B), *E. coli* LPS-induced MIP-1α/CCL3 release occurred at greater than 0.1 ng/mL and was significant at 1 ng/mL (47,464.9 ± 4298.8 pg/mL MIP-1α/CCL3, *n* = 3, *P* < 0.001). *V. vulnificus* LPS-stimulated MIP-1α/CCL3 release, which was statistically significant at 10 ng/mL (18,936 ± 6142 pg/mL MIP-1α/CCL3, *n* = 4, *P* < 0.05), peaked at 100 ng/mL. Thus *V. vulnificus* LPS was a tenth less potent than *E. coli* LPS in inducing MIP-1α/CCL3 release from rat microglia. Interestingly, and in contrast to TNF-α, IL-1-α, IL-6 and MIP-2 release by microglia stimulated by *E. coli* LPS, the effect of *V. vulnificus* LPS on the release of MIP-1α/ CCL3 was *diminished* by 45%. 

The chemokine cytokine-induced neutrophil chemoattractant (CINC)-2α/β or CXCL3, earlier known as growth-regulated oncogene or GRO, plays a role in chemotaxis and inflammation in the CNS [60]. Although human microglia released GRO α *in vitro* [61], the effect of LPS on microglia and CINC-2α/β (CXCL3) generation has not been investigated to our knowledge. As shown in Figure 6 (Panel C), *E. coli* LPS-induced CINC-2α/β (CXCL3) release peaked at 10 ng/mL (16,864 ± 5792 pg/mL CINC-2α/β (CXCL3), *n* = 2, *P* < 0.05). *V. vulnificus* LPS-induced CINC-2α/β (CXCL3) release was statistically significant at 100 ng/mL (27,910 ± 10,443 pg/mL CINC-2α/β (CXCL3), *n* = 2, *P* < 0.01). Thus, similar to TNF-α, IL-1α, IL-6, and MIP-2, *V. vulnificus* LPS was a tenth less potent than *E. coli* LPS in inducing concentration-dependent release of the chemokine CINC-2α/β (CXCL3) *in vitro,* but more effective because maximal release was 65% higher.

The chemokine MCP-1 or CCL2, which plays a role in both neuroinflammation [54] and multiple sclerosis [57], is released by murine [47], rat [48,58] and human microglia *in vitro* [59]. Unstimulated microglia released low levels of MCP-1/CCL2 constitutively (17 ± 9 pg/mL, *n* = 3). As shown in Figure 6 (Panel D), *E. coli* LPS-induced MCP-1/CCL2 release yielding a bell-shaped curve which peaked at 1 ng/mL (441 ± 149 pg/mL MCP-1/CCL2, *n* = 3, *P* < 0.05). Similarly, *V. vulnificus* LPS-induced MCP-1/CCL2 release was statistically significant at 100 ng/mL (506 ± 193 pg/mL MCP-1/CCL2, *n* = 4, *P* < 0.05). Although *V. vulnificus* LPS was a hundredth less potent than *E. coli* LPS at inducing concentration-dependent release of the chemokine MCP-1/CCL2, maximal observed release was similar. 

### 2.7. Effect of V. vulnificus LPS on Rat Brain Microglia TGF-β1 and BDNF

In order to determine whether *V. vulnificus* LPS affected release of anti-inflammatory cytokines and neurotrophins into the conditioned medium [35], we investigated the presence of TGF-β1 and BDNF which have been studied for their neuroprotective effects [62], and have been shown to be expressed constitutively in rat microglia [63], and *in vitro* in *E. coli* LPS-activated human [64] and murine [65] microglia. As shown in Table 1, unstimulated rat microglia released TGF-β1 constitutively (320 ± 50 pg/mL, *n* = 3), but in contrast there was no detectable BDNF. Furthermore, *V. vulnificus* LPS but not *E. coli* LPS significantly enhanced TGF-β1 release from microglia after the 17 h *in vitro* incubation (973.5 ± 264.5 pg/mL TGF-β1, (*n* = 2), *p* < 0.001). No significant increase of BDNF was observed in either *E. coli* or *V. vulnificus* LPS-treated microglia (*n* = 2). 

**Table 1:** The effect of *E. coli* and *V. vulnificus* LPS on rat microglia TGF-β1 and brain-derived neurotrophic factor (BDNF) release.

	TGF-β1 Release				BDNF Release			
LPS	*E. coli*		*V. vulnificus*		*E. coli*		*V. vulnificus*	
(ng/mL)	(pg/mL)	*n* ^1^	(pg/mL)	*n* ^1^	(pg/mL)	*n* ^1^	(pg/mL)	*n* ^1^
0	319.7 ± 50.1	3	319.7 ± 50.1	3	0	2	0	2
0.1	424.3 ± 40.3	3	386 ± 16.0	2	5.1 ± 4.4	2	1.4 ± 0.8	2
1	363.3 ± 36	3	419 ± 66.9	3	18.4 ± 14.9	2	10.2 ± 6.2	2
10	318.7 ± 73.2	3	358 ± 50.1	3	6.9 ± 3.9	2	6.5 ± 5.5	2
100	333.3 ± 58.6	3	310 ± 51.6	3	6.6 ± 3.0	2	9.1 ± 4.7	2
1000	ND		352 ± 55.5	3	ND		7.5 ± 7.5	2
10,000	ND		613 ± 58.7	3	ND		0 ± 0	2
100,000	ND		973.5 ± 264.5 *	2	ND		2.05 ± 2.05	2

^1^ Rat microglia (2 × 10^5^ cells/well) were treated with *E. coli* LPS (0.1–100 ng/mL) or *V. vulnificus* LPS (0.1–10^5^ ng/mL) for 17 h *in vitro*. TGF-β1 and BDNF were determined as described in Experimental Section. Data expressed as pg/mL and is the mean ± SEM of 3 and 2 independent experiments (*n*) for TGF-β1 and BDNF, respectively, each experiment with duplicate determinations. * *P* < 0.001 *versus* untreated control (0).

### 2.8. V. vulnificus LPS Isolation and Chemical Analyses

**Figure 7:**
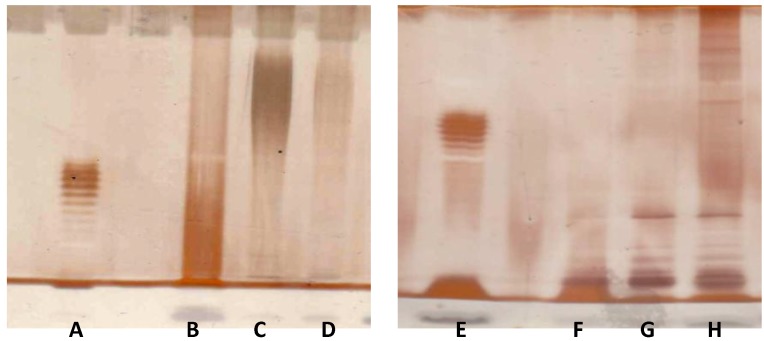
SDS-PAGE of cell wall extracts from *V. vulnificus*. Left panel: Twelve percent gel fixed with Alcian blue prior to silver staining. Right panel: Fifteen percent gel stained directly with the silver nitrate protocol. Lanes **A** and **E**: *E. coli* O55 (8 μg); Lanes **B** and **F**: Petroleum ether–Chloroform–aqueous phenol (PCP) extract (16 μg); Lanes **C** and **G**: Water layer of the phenol/water extraction (16 μg) after enzyme treatment; Lanes **D** and **H**: Water layer of the phenol/water extraction (16 μg) before enzyme treatment.

In order to determine whether the results reported in Figure 1, Figure 2, Figure 3, Figure 4, Figure 5, Figure 6 and Table 1 might reflect a chemical difference between *E. coli vs. V. vulnificus* LPS, *Vibrio vulnificus* strain MO6-24/O cells were extracted according two different methodologies; the extracts were checked by sodium dodecyl sulfate polyacrylamide gel electrophoresis (SDS-PAGE) electrophoresis (Figure 7) and the gels were stained with the silver nitrate protocol. Alcian blue was used as fixative in one case to detect the occurrence of acidic polysaccharide [66]. As expected, lipooligosaccharide (LOS, 162 mg, Figure 7 Lanes B and F) was recovered after PCP (Petroleum ether–Chloroform–aqueous phenol) extraction of cells. The remaining pellet was extracted according to the hot water/phenol protocol. The silver stained SDS-PAGE profile of the crude extract of the water layer (Figure 7, Lane H) contained a dense banding spread throughout the gel, while comparison of the same sample stained applying Alcian blue fixative first suggested the occurrence of a polysaccharide (PS) in the upper part of the gel (Figure 7, Lane D). To confirm this hypothesis, the crude sample (219 mg) was treated with DNAse, RNAse and proteinase K; SDS-PAGE control of the purified sample (79 mg) indeed showed that the dense banding was lost (Figure 7, Lane G) while the use of the fixative gave a positive and intense staining of the PS (Figure 7, Lane C). In addition, SDS-PAGE profiles showed that LOS was pure and not contaminated from the acidic polysaccharide, while minimal traces of LOS were seen in the water extracts.

**Figure 8:**
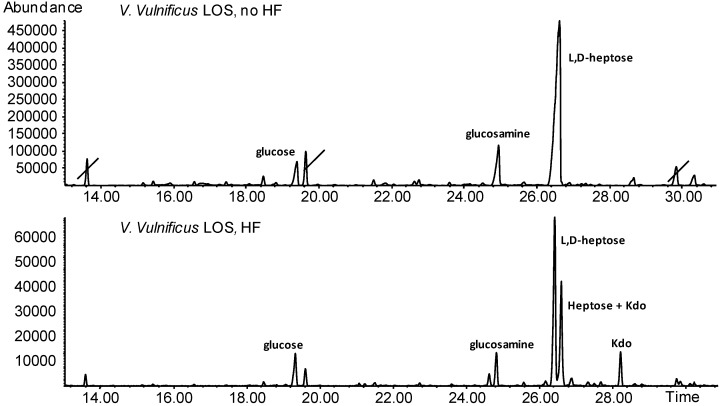
Gas chromatography-mass spectrometry (GC-MS) chromatograms of the acetylated methyl glycosides of the lipooligosaccharide (LOS) fraction from *V. vulnificus*. The upper profiles indicate the monosaccharide composition of the LOS without hydrogen fluoride (HF) treatment and does not contain 2-keto-3-deoxy-d-*manno*-octulosonic acid (Kdo); the low profile is obtained after HF treatment of the sample. Peaks barred are impurities.

Further information on the chemical constituents of both LOS and PS from *V. vulnificus*, came from gas chromatography–mass spectrometry (GC-MS) analysis of the corresponding acetylated methyl glycosides. As for the LOS (Figure 8, upper panel), composition analysis detected the occurrence of l-*glycero*-d-*manno*-heptose, glucosamine and glucose but not Kdo (2-keto-3-deoxy-d-*manno*-octulosonic acid), the hallmark monosaccharide of LOS. Therefore, suspecting the presence of phosphate on this residue, LOS was treated with aqueous hydrogen fluoride (HF) and the analysis repeated (Figure 8, lower panel). The chromatogram contained two additional peaks, of which the one at 28.2 min was the main peak of Kdo, while the one at 26.2 min was another type of heptose together with the minor peak produced from Kdo during this chemical procedure. Lipid composition was inferred by GC-MS analysis as well and showed the presence of C12:0 3-OH, C14:0, C14:0 2-OH and C14:0 3-OH (data not shown).

**Figure 9:**
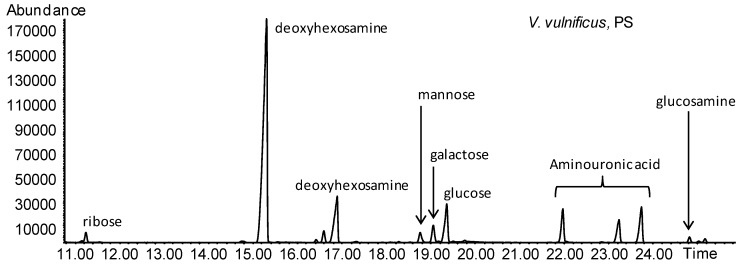
GC-MS chromatograms of the acetylated methyl glycosides of *V. vulnificus* polysaccharide (PS). Ribose peak is a reminiscence of the RNA contaminating the sample.

As for the PS composition (Figure 9), residues characteristic of the LOS were below the detection limit, even when HF treatment was performed (data not shown). This polysaccharide contained mainly a 6-deoxy-hexosamine residue and an amino uronic acid residue. The ring stereochemistry of both these monosaccharides could not be determined at this stage because of the lack of appropriate standards and will be the object of further work.

## 3. Discussion

The role of microglia activation in central nervous system infections [25] as well as the involvement of O_2_^−^ generation in the mechanism of neuroinflammation and neurodegeneration has received considerable attention over the past two decades [26,30,67]. One significant activator of microglia is LPS [68], which may activate microglia via the lipid A portion of the macromolecule, and then stimulate release of O_2_^−^ as well as additional proinflammatory mediators such as matrix metalloproteinases, arachidonic acid metabolites, cytokines and chemokines [26]. 

The first aim of our investigation was to test the hypothesis that LPS purified from human clinical *V. vulnificus* strain MO6-24/O might activate rat brain microglia and result in O_2_^−^ release *in vitro*. Our experimental data lends support to our working hypothesis and the following deductions: First, as previously reported [27,38], a 17 h incubation of microglia with *E. coli* LPS resulted in an initial dose-dependent enhancement of PMA-stimulated O_2_^−^ release followed by a progressive inhibition at greater than 1 ng/mL, which paralleled an increase in LDH release. Second, *V. vulnificus* MO6-24/O LPS-treated microglia generated O_2_^−^ as depicted by a bell-shaped dose-response curve that in contrast to *E. coli* LPS peaked at 10 ng/mL, whereupon a progressive attenuation of O_2_^−^ generation concomitant to increased LDH release was observed. Third, the observed right shift of the bell-shaped dose-response curve depicting O_2_^−^ generation by microglia treated with *V. vulnificus* MO6-24/O LPS indicates that this particular *V. vulnificus* LPS was less potent than *E. coli* LPS. Future studies should be undertaken to determine whether peak O_2_^−^ generation by *V. vulnificus* MO6-24/O LPS require 18-24 h to develop, as observed with *E. coli* LPS-treated microglia [27].

Our second aim was to investigate whether concomitant with O_2_^−^ generation, *V. vulnificus* MO6-24/O LPS-treated microglia might release pro-inflammatory mediators, as we have observed with both *E. coli* LPS [27] and, more recently, with *M. aeruginosa* LPS-treated microglia [38]. Our data supports the following observations: First, confirming the above mentioned studies, after a 17 h *in vitro* incubation with *E. coli* LPS, microglia released both TXB_2_ and MMP-9, as well as the following cytokines and chemokines in the following rank order: MIP-2/CXCL2 > MIP-1α/CCL3 > CINC-2α&β/CXCL3 > IL-6 > TNF-α > IL-1α > MCP-1/CCL2. Second, and for the first time to our knowledge, concomitant with significant O_2_^−^ release, *V. vulnificus* MO6-24/O LPS-treated microglia generated not only TXB_2_ and MMP-9, but also proinflammatory cytokines and chemokines in the following rank order: MIP-2/CXCL2 > CINC-2α & β/CXCL3 > IL-6 > MIP-1α/CCL3 > TNF-α > IL-1α > MCP-1/CCL2. Third, similar to O_2_^−^ generation, and supporting the notion that *V. vulnificus* MO6-24/O LPS was less potent but more effective than *E. coli* LPS, with the exception of MIP-1α, all the pro-inflammatory mediators investigated were released in larger quantities *in vitro*. Fourth, the progressive *in vitro* attenuation of PMA-elicited O_2_^−^ generation in *V. vulnificus* MO6-24/O LPS-treated microglia correlated with both the concentration-dependent LDH release as well as the presence of TXB_2_, MMP-9, and the cytokines and chemokines that were investigated. Our current study suggests, but does not conclusively prove, that the studied proinflammatory mediators may contribute to the mechanism of *V. vulnificus* MO6-24/O LPS-induced cytotoxicity to microglia *in vitro*. This intriguing possibility remains to be investigated in future studies.

It is important to reflect on several potential new lines of inquiry that have emerged from the observed effects of *V. vulnificus* MO6-24/O LPS on rat neonatal microglia *in vitro*. First, because the present study was completed with *V. vulnificus* MO6-24/O LPS, an archetypical clinical *V. vulnificus* strain [7,8], it would be important to determine whether LPS isolated from other *V. vulnificus* strains, particularly those from the environment, would also be bioactive in the *in vitro* rat microglia model*.* Second, because our experimental paradigm used *neonatal* brain microglia, an important next study would be to determine whether *V. vulnificus* MO6-24/O LPS might activate *adult* rat microglia, which release higher levels of PGE_2_ than neonatal microglia [69], and may perhaps differ in their capacity to generate O_2_^−^, as well as other inflammatory mediators. Third*,* determining whether treatment of *human* microglia with *V. vulnificus* LPS *in vitro* would also show an *in vitro* biphasic O_2_^−^ generation should be investigated, because *E. coli* LPS would prime human microglia O_2_^−^ release *in vitro* [70]. Fourth, *in vivo* studies should be undertaken to determine whether systemic inflammation caused by *V. vulnificus* MO6-24/O LPS may be pathogenic to the brain immune system, perhaps being less potent but more efficacious than *E. coli* LPS, as we have observed in our *in vitro* studies, because this *V. vulnificus* biotype has been shown to be lethal to mice [14] and induce cytokine release from human peripheral blood leukocytes [19]. The induction of reactive oxygen species by *V. vulnificus* LPS is also intriguing in light of recent work suggesting that the inflammatory response is attenuated in peripheral blood mononuclear cells from chronic alcohol users with evidence of oxidative stress following *in vitro* exposure to live *V. vulnificus* bacterial cells [71]. Fifth, further studies on the chemical structure of *V. vulnificus* LPS are necessary to determine its potential relationship with the observed bioactivity in this study. We are hopeful that further investigation of the immunotoxicology of *V. vulnificus* LPS on brain microglia both *in vitro* and *in vivo* will contribute to the development of novel therapeutic strategies to protect and treat humans exposed to both clinical and environmental sources of *V. vulnificus* strains. 

## 4. Experimental Section

### 4.1. Reagents

*Escherichia coli* LPS (*E. coli*) (O26:B6) was from Difco Laboratories, Detroit, MI, USA, and *V. vulnificus* LPS (1.075 × 10^5^ endotoxin units/mg), prepared from *V. vulnificus* strain MO6-24/O LPS as described [19], was used in experiments depicted in Figure 1, Figure 2, Figure 3, Figure 4, Figure 5, Figure 6 and Table 1. Dulbecco’s modified Eagle medium (DMEM) with high glucose (4500 mg/L), Hanks’ balanced salt solution (HBSS), penicillin (P), streptomycin (S) and trypsin (0.25%)-EDTA (1 mM) were from GIBCO Laboratories, Life Technologies Inc., Grand Island, NY, USA; heat-inactivated fetal bovine serum certified (FBS) was from Hyclone, Logan, UT, USA; ferricytochrome c type III (from horse heart) (FCC), superoxide dismutase (from bovine liver) (SOD), phorbol 12-myristate 13-acetate (PMA) were from Sigma Chemical Co., St. Louis, MO, USA. PMA was maintained at −20 °C as a 8 mM stock solution in DMSO.

### 4.2. LPS Contamination

All glassware and metal spatulas were baked for 4 h at 210 °C to inactivate LPS [72]. Sterile and LPS-free 225 cm^2^ vented cell culture flasks were from BD Biosciences, San Jose, CA, USA; 24-well flat-bottom culture clusters were from Costar^®^, Corning Inc., Corning, NY, USA; disposable serological pipettes were from Greiner Bio-One, Monroe, NC, USA. Sterile and pyrogen-free Eppendorf Biopur pipette tips were from Brinkmann Instruments, Inc., Westbury, NY, USA. 

### 4.3. Isolation of Rat Neonatal Microglia

Experiments were performed in adherence to National Institutes of Health guidelines on the use of experimental animals, with protocols approved by Midwestern University's Research and Animal Care Committee. Rat brain neonatal microglia were isolated and characterized as previously described [27]. Briefly, cerebral cortices of 1–2 day-old Sprague-Dawley rats from Charles Rivers Laboratories, Portage, MI, USA, were surgically removed, placed in cold DMEM containing 120 U/mL P and 12 μg/mL S, the meninges removed, and brain tissue minced and dissociated with trypsin-EDTA at 35.9 °C for 3–5 min. The mixed glial cell suspension was plated in 225 cm^2^ vented cell culture flasks with DMEM medium supplemented with 10% FBS containing 120 U/mL P and 12 μg/mL S, and grown in a humidified 5% CO_2_ incubator at 35.9 °C for 12–14 days. Upon confluence (Day 14) and every week thereafter, microglia were detached using an orbital shaker (150 rpm, 0.5 h, 35.9 °C, 5% CO_2_), centrifuged (400× *g*, 25 min, 4 °C), and cell number and viability assessed by trypan blue exclusion. In our laboratory, rat neonatal microglia yields averaged 1.1 × 10^6^ microglia per tissue culture flask (225 cm^2^) per week. Depending on the particular experimental design (see below), microglia averaging > than 95% viability were plated in 24-well cell culture clusters, with DMEM supplemented with 10% FBS containing 120 U/mL P and 12 μg/mL S, and placed in a humidified 5% CO_2_ incubator at 35.9 °C 24 h prior to the experiments.

### 4.4. Activation of Microglia with LPS (Experimental Design)

To determine the *in vitro* effect of *V. vulnificus* MO6-24/O LPS on rat neonatal microglia activation and inflammatory mediator release (O_2_^−^, eicosanoids, matrix metalloproteinases, cytokines, and chemokines), 2 × 10^5^ rat neonatal microglia were seeded in DMEM + 10% FBS + 120 U/mL P + 12 μg/mL S into each well of nonpyrogenic polystyrene 24-well flat-bottom culture clusters (Costar^®^, Corning Inc., Corning, NY, USA), and stimulated with 0.1–10^5^ ng/mL *V. vulnificus* LPS for 17 h in a humidified 5% CO_2_ incubator at 35.9 °C. *E. coli* LPS (0.1–100 ng/mL) was used as a control in all the experiments described herein [27]. After the 17h incubation, conditioned media (1 mL) from each tissue culture well was aspirated and split into two aliquots. One aliquot (0.1 mL) was used to measure lactic dehydrogenase (LDH) levels, as a measure of cell viability [39]. The remaining aliquot (0.9 mL) was frozen (−84 °C) until determination of eicosanoids, cytokines, chemokines, and matrix metalloproteinases, as described below. Once the conditioned media had been removed, both *V. vulnificus* and *E. coli* LPS–treated microglia cells were washed with warm (37 °C) HBSS, and O_2_^−^ was determined as described below.

### 4.5. Assay for Superoxide Anion (O_2_^−^) Generation

O_2_^−^ generation was determined by the superoxide dismutase (SOD)-inhibitable reduction of ferricytochrome C (FCC) [27]. Briefly, PMA (1 μM)-triggered O_2_^−^ release from either *E. coli* or *V. vulnificus* LPS-activated microglia was measured in the presence of FCC (50 μM) and HBSS, with or without SOD (700 Units), which inhibited >95% of FCC reduction, during a 70 min incubation in a humidified 5% CO_2_ incubator at 35.9 °C. All experimental treatments were run in duplicate and in a final volume of 1 mL. Changes in FCC absorbance were measured at 550 nm using a Beckman DU-800 spectrophotometer. Differences in the amount of reduced FCC in the presence and absence of SOD were used to determine microglia O_2_^−^ generation by employing the molecular extinction coefficient of 21.0 × 10^3^ M^−1^ cm^−1^ and expressed in nmol. 

### 4.6. Assay for Lactic Dehydrogenase (LDH)

To assess cell viability of microglia treated with either *V. vulnificus* LPS or *E. coli* LPS as described in our experimental design, conditioned media was harvested following preincubation and LDH release was determined as described [27,39]. Microglia LDH release was expressed as a percent of total LDH released by 0.1% Triton X-100-lysed microglia. Because the fetal bovine serum contained LDH (data not shown), unless LDH release from LPS-treated microglia was greater than 15% of that observed from Triton X-100 (0.1%)-treated microglia (total LDH), LPS treatment was considered to have had no effect on microglia viability. 

### 4.7. Assay for Thromboxane B_2_ (TXB_2_) Generation

Following incubation of microglia with either *V. vulnificus* LPS or *E. coli* LPS for 17 h, TXB_2_ generation in cell-free conditioned media was measured using a TXB_2_ immunoassay (Cayman Chemical, Ann Arbor, MI, USA), as indicated in the manufacturer’s protocol. Results were expressed as picogram per mL (pg/mL). The minimum detectable concentration was 7.8 pg/mL TXB_2_.

### 4.8. Gelatinase Zymography for MMP-2 and MMP-9 Analysis

Gelatin-containing zymograms were used to detect MMP-2 (68 kDa) and MMP-9 (92 kDa) and their identification was based on molecular weight. Following incubation of cultured rat neonatal microglia with either *V. vulnificus* LPS or *E. coli* LPS, MMP-2 and -9 release were determined in the cell-free conditioned media. As the rat neonatal microglia cultures were normalized for cell number, equal volumes of harvested media obtained from each condition were analyzed. Briefly, 90 μg of each protein sample were electrophoresed using a 10% polyacrylamide gel containing 0.1% gelatin. The gels were then incubated twice for 30 min in 1× Novex Zymogram Renaturing Buffer (Invitrogen, Carlsbad, CA, USA), incubated overnight in a 5% CO_2_ incubator at 37 °C, and stained in 0.4% (wt/vol) Coomassie Brilliant Blue R-250 Solution (Bio-Rad, Hercules, CA, USA). Sequential destaining first in 40% methanol, 10% acetic acid, and then in 10% methanol, 10% acetic acid allowed MMP activity to be visualized as clear bands against a blue background. Images of zymograms were obtained using a Kodak Gel Logic 1500 Imaging System and Molecular Imaging Software (Kodak, Rochester, NY, USA). Semiquantitation of zymograms was performed using the UN-SCAN-IT™ gel automated digitizing system from Silk Scientific (Orem, UT, USA). Microglia MMP release was normalized between experiments by dividing values in pixels for treated samples by their respective controls.

### 4.9. Assays for Cytokines: TNF-α, IL-1α, IL-6 and TGF-β1

The presence of immunoreactive cytokines TNF-α, IL-1α, IL-6, TGF-β1 in the cell-free conditioned media was determined using rat-specific ELISAs from Biosource International (Camarillo, CA, USA). The results were expressed in pg/mL. The minimum detectable cytokine concentrations were: TNF-α, less than 4 pg/mL; IL-1α, less than 3 pg/mL; IL-6, less than 7 pg/mL and TGF-β1, less than 15.6 pg/mL. 

### 4.10. Assays for Chemokines: MIP-1α/CCL3, MIP-2/CXCL2, MCP-1/CCL2 and CINC-2α/β/CXCL3

The presence of immunoreactive chemokines in cell-free conditioned media was determined using rat-specific ELISAs: For MIP-1α/CCL3 from Koma Biotech, Seoul, South Korea; for MIP-2/CXCL2 and MCP-1/CCL2 from Biosource International, Camarillo, CA, USA, and for CINC-2α/β/CXCL3 from R & D Systems, Minneapolis, MN, USA. The results were expressed in pg/mL. The minimum detectable chemokine concentrations were: MIP-1α/CCL3, less than 16 pg/mL; MIP-2/CXCL2, less than 1 pg/mL; MCP-1/CCL2, less than 8 pg/mL; and CINC-2α/β/CXCL3, less than 0.8 pg/mL.

### 4.11. Assay for the Neurotrophin Brain Derived Neurotrophic Factor (BDNF)

BDNF generation in cell-free conditioned media was measured using a rat-specific ELISA for BDNF from EMD Millipore, Billerica, MA. The results were expressed in pg/mL. The minimum detectable concentration was less than 7.8 pg/mL. 

### 4.12. V. vulnificus LPS Chemical Analyses

*V. vulnificus* strain MO6-24/O LPS was streaked from frozen stocks onto LB agar medium and incubated at 37 °C overnight. Bacteria from single colonies were inoculated into 10 mL LB broth and shaken overnight at 37 °C. Two mL of the overnight culture were spread onto each of five 21.5 × 27 cm pans containing approximately 250 mL of LB agar medium and incubated overnight at 37 °C. Cells were harvested from the trays, suspended in 15 mL deionized water, and heat shocked at 70 °C for 7 min. The cell suspension was then frozen and lyophilized. *V. vulnificus* LPS was prepared for chemical analysis from the *V. vulnificus* strain MO6-24/O LPS as described [73]. LPS monosaccharides and lipids were analysed as acetylated *O*-methyl glycosides and methylesters, respectively, as described [66]. Dephosphorylation was carried out keeping LPS (0.5 mg) in aqueous HF (50%, 50 μL) at room temperature overnight. The solution was evaporated under a stream of air and the dried material was analysed after transformation into the corresponding acetylated methyl glycosides. GC-MS analyses were performed with an Agilent 6850 coupled to MS Agilent 5973, equipped with a SPB-5 capillary column (Supelco, 30 m × 0.25 mm *i.d.*, flow rate, 0.8 mL min^−1^) and He as carrier gas. Electron impact mass spectra were recorded with an ionization energy of 70 eV and an ionizing current of 0.2 mA. The temperature program used for the analyses was the following: 150 °C for 5 min, 150→300 °C at 3 °C/min, 300 °C for 5 min.

### 4.13. Statistical Analysis

Data was expressed as mean ± SEM from two to four independent experiments (*n*), each experiment with triplicate determinations. Data were analyzed with Prism^®^ software package Version 6 from GraphPad, San Diego, CA, USA. LPS-treated microglia were compared with the vehicle-treated microglia (control), shown as zero in the corresponding figures. One-way ANOVA followed by Dunnett’s *post hoc* procedure was performed on all sets of data. Statistical significance between the effect of a single dose of *E. coli* and *V. vulnificus* LPS on the generation of each mediator investigated (e.g., O_2_^−^) was determined using two-way ANOVA. Differences were considered statistically significant at *p* < 0.05 and reported in each figure legend. 

## 5. Conclusions

Concentration-dependent treatment of neonatal brain microglia with *V. vulnificus* MO6-24/O LPS resulted in a significant rise in O_2_^−^ production, followed by progressive decrease in O_2_^−^ release, concomitant with release of LDH, and generation of TXB_2_, MMP-9, cytokines and chemokines. We hypothesize that the inflammatory mediators investigated may be involved in the mechanism of injury to microglia *in vitro*, by an as yet undetermined autocrine mechanism. Although *in vitro V. vulnificus* LPS was less potent than *E. coli* LPS, inflammatory mediator release was clearly more efficacious. A possible explanation for this result is that the *in vitro* microglia system is able to perceive chemical differences between *V. vulnificus* and *E. coli* LPS. Our chemical data show that Kdo in *V. vulnificus* LPS is fully phosphorylated and that the fatty acid pattern is similar with the exception of C14:0 2-OH which is absent in *E. coli.* These differences may be of importance in the modulation of the biological activity by LPS and more insights will probably be gained after the complete structure of *V. vulnificus* LPS is elucidated.

Finally, we hypothesize that should *V. vulnificus* LPS gain entry into the CNS, it is possible that microglia may become activated, resulting in high levels of O_2_^−^ release as well as neuroinflammatory TXB_2_, MMP-9, cytokines and chemokines. *In vivo* studies with *V. vulnificus* LPS will be required to test this intriguing hypothesis. 

## Abbreviations

BDNF: brain derived neurotrophic factor
CINC-2α/β/CXCL3: cytokine-induced neutrophil chemoattractant or macrophage inflammatory protein-2b or chemokine ligand 3
DMEM: Dulbecco’s modified Eagle medium
*E. coli*: *Escherichia coli*
FBS: fetal bovine serum certified
FCC: ferricytochrome c type III
HBSS: Hank’s balanced salt solution
IL: interleukin
LDH: lactic dehydrogenase
LPS: lipopolysaccharide
MCP-1/CCL2: monocyte chemotactic protein-1 or chemokine ligand 2
MIP-1α/CCL3: macrophage inflammatory protein 1α or chemokine ligand 3
MIP-2/CXCL2: macrophage inflammatory protein-2
MMP-9: matrix metalloproteinase-9
O_2_^−^: superoxide anion
P: penicillin
PBS: phosphate buffered saline
PMA: phorbol 12-myristate 13-acetate
S: streptomycin
SEM: standard error of the mean
SOD: superoxide dismutase
TGF-β1: transforming growth factor β1
TNF-α: tumor necrosis factor
TXB_2_: thromboxane B_2_
*V. vulnificus*: *Vibrio vulnificus*
